# Supplementary material for: An Updated Structure of Oxybutynin Hydrochloride
Source: bioRxiv. 2024 Jun 6:2024.06.05.597682. Preprint. [Version 1] doi: 10.1101/2024.06.05.597682 (PMC11185708; doi:10.1101/2024.06.05.597682)
Supplement: Supplement 1 [file media-1.pdf]

Supporting Information

An Updated Structure of Oxybutynin Hydrochloride

Jieye Lin[1], Guanhong Bu[1], Johan Unge[2] and Tamir Gonen[1,3,4]*

[1] Department of Biological Chemistry, University of California, Los Angeles, 615 Charles E. Young Drive South, Los Angeles, California 90095, United States

[2] Department of Chemistry, Umeå University, 901 87 Umeå, Sweden

[3] Department of Physiology, University of California, Los Angeles, 615 Charles E. Young Drive South, Los Angeles, California 90095, United States

[4] Howard Hughes Medical Institute, University of California, Los Angeles, Los Angeles, California 90095, United States

* Corresponding Author T.G. tgonen@g.ucla.edu

Methods

Materials.

Oxybutynin hydrochloride, 4-(diethylamino)but-2-ynyl (*R*/*S*)-2-cyclohexyl-2-hydroxy-2-phenyl-acetate hydrochloride, was commercially purchased from InvivoChem and used as received, without further recrystallization.

Grid preparation.

The sample preparation followed the procedure as described previously.[1] One carbon-coated copper grid (200-mesh, 3.05 mm O.D., Ted Pella Inc.) was pretreated with 15 mA negative glow-discharge plasma for 60 s using PELCO easiGlow (Ted Pella Inc.). Less than 1 mg of powdery compounds was mixed with the grid in a 10 mL scintillation vial with 30 s gentle shaking. The grid was clipped outside using c-ring and autogrid clip at room temperature.

MicroED data collection.

The clipped grid was loaded in Thermo Fisher Talos Arctica Cryo-TEM (200 kV, ~0.0251 Å), which is equipped a CMOS CetaD camera (4096 × 4096 pixels) and EPUD software (Thermo Fisher).[2] Screening of microcrystals was done in the imaging mode (SA 3400×). Only the thin crystals with a certain brightness contrast were selected. Their eucentric heights were calibrated to maintain the crystals inside the beam during the continuous rotation. The MicroED data was collected in the diffraction mode at the camera length of 741 mm (the calibrated sample-detector distance)

and microprobe beam size 11 under the parallel beam condition (45.2% C2 intensity). A 70 µm C2 aperture and a 50 µm selected area (SA) aperture were used to result in an approximately 1.4 µm width beam area (0.0098 $e^{-1}/Å^2/s$).[3] Typical data collection used a constant rotation rate of 2° per second over an angular wedge of 130° from -65° to +65°, with 0.5s exposure time per frame, resulting a total dose of 0.65 $e^{-}/Å^2$ for each dataset.

MicroED data processing.

MicroED data was saved in mrc format and converted to smv format using the mrc2smv software (https://cryoem.ucla.edu/microed).[2] The converted frames were indexed and integrated by XDS.[4,5] One dataset with the highest resolution (0.87 Å) was scaled using XSCALE to achieve 83.9% completeness.[5] Intensities were converted to SHELX hkl format using XDSCONV[5] and *ab initio* solved by SHELXT.[6] The structure was refined by SHELXL[7] using Shelxle[8] as a graphical interference to yield the final MicroED structure (Table S1).

Molecular Docking

The ligand structures from MicroED structure **1R** and X-ray structure **2** (CSD entry: MBCHPA)[9] and **4** (CSD entry: IPILUQ)[10] were extracted. The amine hydrogens were removed based on pKa value.[11] All the water and Cl^{-} anions were removed. The ligand structures were imported to AutoDock Tools 1.5.7,[12] making all active torsion bonds rotatable (Figure S4). The protein structure was downloaded from PDB (Protein Data Bank, https://www.rcsb.org/) entry 4U15.[13] Ligands, ions, water were removed using Pymol 2.5.5.[14] The resulting protein structure was modified by adding hydrogen atoms and charges as computed by AutoDock Tools 1.5.7.[12]

The protein cavity was analyzed by CB-Dock2 webtool[15] at the center of x,y,z = (17, 85, 56), which is consistent with the orthosteric binding site observed in PDB structure 4U15.[13] A grid box measuring 18.75 Å × 18.75 Å × 18.75 Å with 0.375 Å spacing was positioned. During the docking in AutoDock Vina 1.1.2,[16,17] the ligands were set to be flexible (Figure S4), while the protein was treated with rigid model. The docked complex with minimum binding energy was analyzed in Protein-Ligand Interaction Profiler (PLIP) web tool and Pymol 2.5.5 (Figure 3 and Tables S3-S6).[14,18]

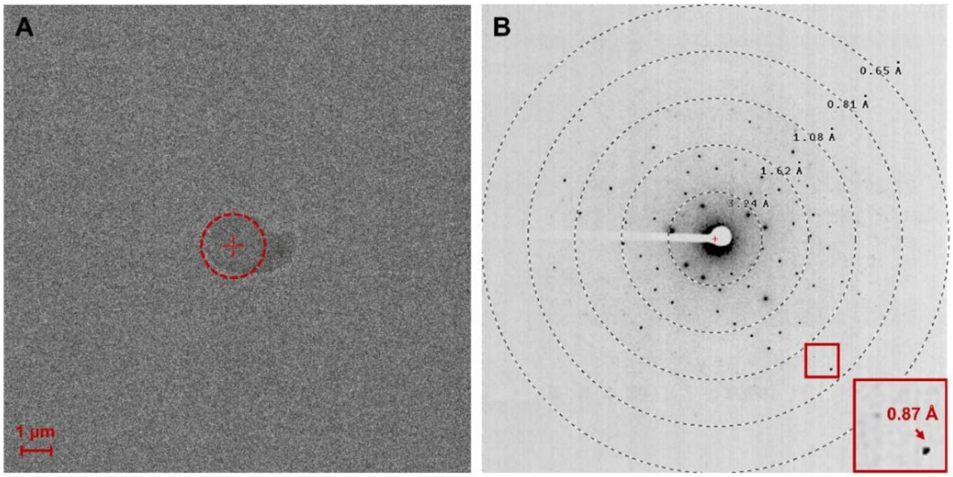

Figure S1 Crystal appearance and diffraction pattern under the TEM. (A) Image of oxybutynin hydrochloride **1** under the imaging mode (SA 3400×). The diffraction beam area was highlighted in dashed red circles; (B) Diffraction pattern of oxybutynin hydrochloride **1** under diffraction mode (741 mm).

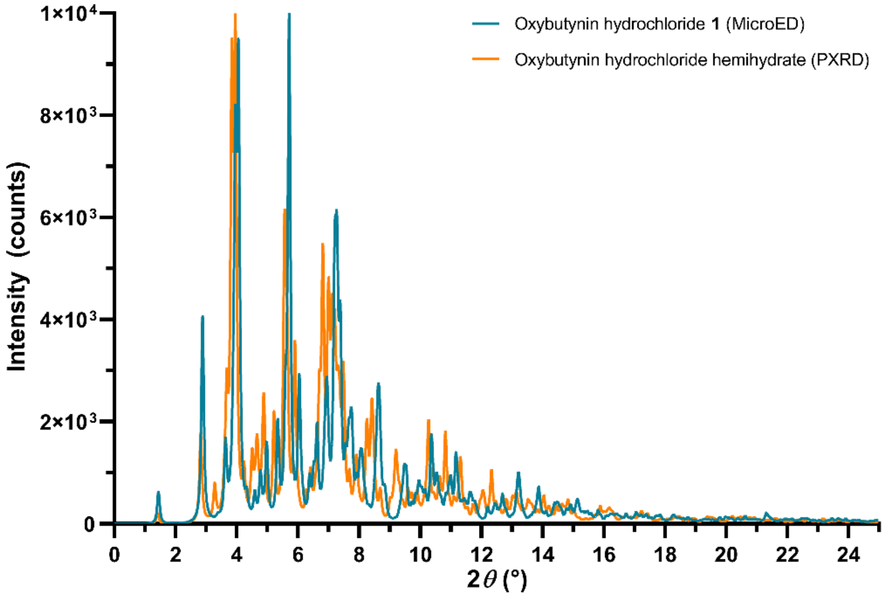

Figure S2 Overlay of PXRD patterns of oxybutynin hydrochloride **1** and the literature reported oxybutynin hydrochloride hemihydrate.[19] The PXRD patterns were back-calculated using the MicroED and PXRD structures in Mercury software[20] (wavelength was set as 0.457667Å with 0.001° 2θ step to be consistent with literature[19]), colored in green and orange, respectively.

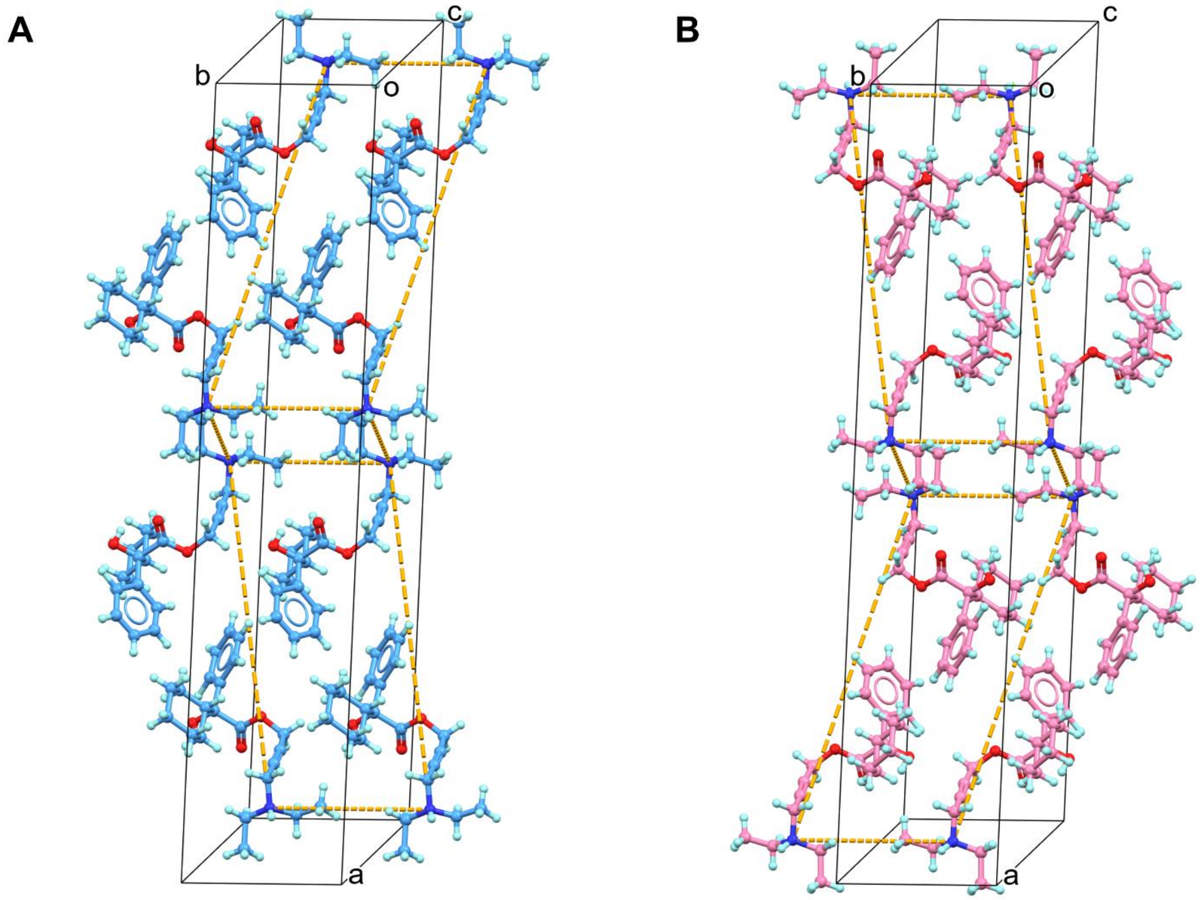

Figure S3 (A) Packing diagram of **1R** layers; (B) Packing diagram of **1S** layers. **1R** was colored in blue, **1S** was colored in violet. Cl^- anions were omitted for clarification. The dashed lines in orange indicated the different positions **1R** and **1S** occupied in the unit cell.

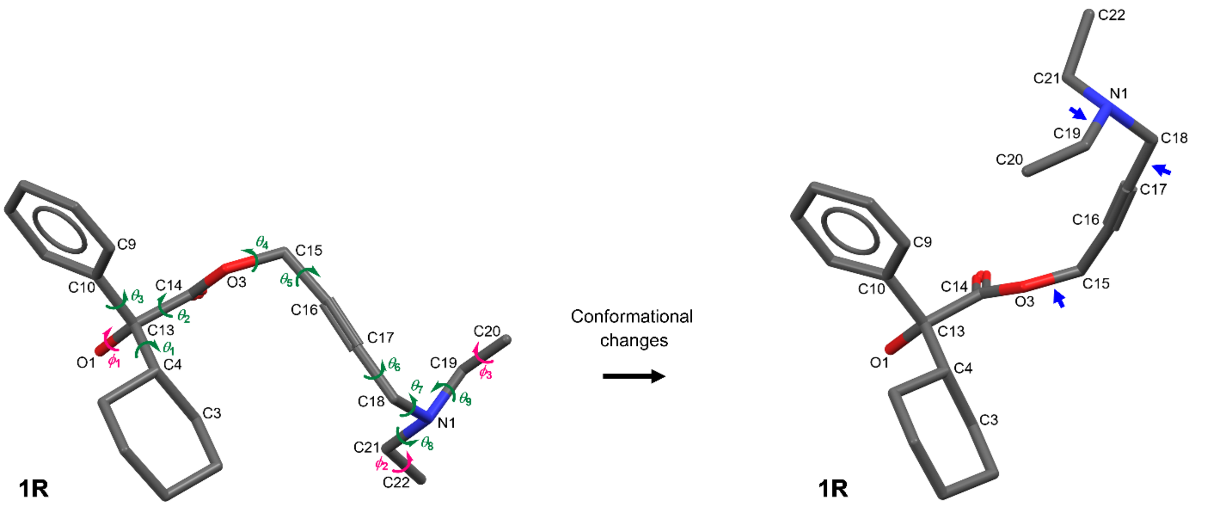

Drug formulation state	*Biologically active state*
θ_1 **(C3–C4–C13–O1)** : 61.16°	θ_1 **(C3–C4–C13–O1)** : 48.81°
θ_2 **(O3–C14–C13–O1)** : -178.89°	θ_2 **(O3–C14–C13–O1)** : -157.76°
θ_3 **(C9–C10–C13–O1)** : 162.08°	θ_3 **(C9–C10–C13–O1)** : 171.7°
θ_4 **(C14–O3–C15–C16)** : -89.25°	θ_4 **(C14–O3–C15–C16)** : 140.17°
θ_5 **(O3–C15–C16–C17)** : 25.59°	θ_5 **(O3–C15–C16–C17)** : -37.00°
θ_6 **(C16–C17–C18–N1)** : -128.98°	θ_6 **(C16–C17–C18–N1)** : 144.13°
θ_7 **(C17–C18–N1–C19)** : 56.33°	θ_7 **(C17–C18–N1–C19)** : 67.58°
θ_8 **(C18–N1–C21–C22)** : -170.32°	θ_8 **(C18–N1–C21–C22)** : -92.06°
θ_9 **(C18–N1–C19–C20)** : 58.37°	θ_9 **(C18–N1–C19–C20)** : -106.85°

Figure S4 The conformation changes of **1R** from drug formulation state to biologically active state**.** θ_1–θ_9 are colored in green which dramatically influence the whole structure; ϕ_1–ϕ_3 are colored in red that have less impact on the overall structure. The major changes were marked with blue arrows.

Table S1 MicroED data statistics of oxybutynin hydrochloride **1**.

Stoichiometric formula	$C_{22}H_{32}ClNO_3$
Molar mass	393.94
Temperature (K)	80
Crystal system	Monoclinic
Space group	C 2/c
Unit cell lengths (Å)	
a	38.85
b	7.92
c	14.43
Unit cell angles (°)	
α	90.000
β	110.560
γ	90.000
Cell volume (Å^3)	4157.19
No. of observed reflections	9001
No. of unique reflections	3004
R_{obs} (%)	15.9
R_{meas} (%)	19.4
I/Sigma	4.50
$CC_{1/2}$	98.8
**Resolution (Å)**	**0.87**
**Completeness (%)**	**83.9**
**R_1 (%)**	**18.42**
wR_2 (%)	45.40
GooF	1.442

Table S2 Hydrogen bonds statistics of oxybutynin hydrochloride **1** (Å, °).

**1R**	**D–H**	**H···A**	**D···A**	**D–H···A**
N1–H···Cl1	1.139	1.959	3.049	158.78
O1–H···Cl1′	0.733	2.590	3.085	126.69
C19–H···Cl1′	0.972	2.714	3.592	150.51
**1S**	**D–H**	**H···A**	**D···A**	**D–H···A**
N1′–H···Cl1′	1.139	1.959	3.049	158.78
O1′–H···Cl1	0.733	2.590	3.085	126.69
C19′–H···Cl1	0.972	2.714	3.592	150.51

Table S3 Protein-ligand interactions in M_3R/**1R** complex predicted by molecular docking (Å, °).

Salt Bridges				
**Residue**	**AA**	**Distance**	**Protein positive?**	**Ligand Group[a]**
147	ASP	3.59	No	DEA

Hydrogen Bonds					
**Residue**	**AA**	**Ligand Group[a]**	**H–A**	**D–A**	**D–H···A**
151	SER	DEA	3.20	4.10	157.61
507	ASN	Carb	2.05	2.94	144.19

π-stacking					
**Residue**	**AA**	**Ligand Group[a]**	**Distance[a]**	**Angle[b]**	**Stacking Type**
503	TRP	Ph	4.91	69.85	T-shaped

Hydrophobic Interactions			
**Residue**	**AA**	**Ligand Group[a]**	**Distance**
148	TYR	DEA	3.59
148	TYR	Cy	3.84
152	ASN	Ph	3.53
155	VAL	Ph	3.68
199	TRP	Cy	3.49
231	THR	Cy	3.65
234	THR	Cy	3.79
235	ALA	Cy	3.95
238	ALA	Ph	3.68
503	TRP	DEA	3.71
506	TYR	ALip	3.77
529	TYR	ALip	3.88
533	TYR	DEA	3.52

Notes: [a]Ligand group was represented by abbreviation: diethylamine (DEA), carbonyl group (Carb), phenyl group (Ph), cyclohexyl group (Cy), aliphatic chain (ALip); [b]Distances were measured between centroids determined as the center of aromatic rings. [c]Angles were measured by the angles between planes of two aromatic rings.

Table S4 Protein-ligand interactions in M_3R/**2** complex predicted by molecular docking (Å, °).

Salt Bridges					
**Residue**	**AA**	**Distance**	**Protein positive?**	**Ligand Group[a]**	
147	ASP	3.42	No	DMA	
**Hydrogen Bonds**					
**Residue**	**AA**	**Ligand Group[a]**	**H–A**	**D–A**	**D–H···A**
151	SER	DMA	3.08	3.97	156.92
507	ASN	Carb	1.99	2.89	145.07
**π-stacking**					
**Residue**	**AA**	**Ligand Group[a]**	**Distance[b]**	**Angle[c]**	**Stacking Type**
503	TRP	Ph	4.81	62.68	T-shaped
**Hydrophobic Interactions**					
**Residue**	**AA**	**Ligand Group[a]**	**Distance**		
148	TYR	Cy	3.61		
152	ASN	Ph	3.57		
155	VAL	Ph	3.77		
199	TRP	Cy	3.54		
231	THR	Cy	3.63		
238	ALA	Ph	3.74		
506	TYR	Cy	3.82		
529	TYR	ALip	3.73		

Notes: [a]Ligand group was represented by abbreviation: dimethylamine (DMA), carbonyl group (Carb), phenyl group (Ph), cyclohexyl group (Cy), aliphatic chain (ALip); [b]Distances were measured between centroids determined as the center of aromatic rings. [c]Angles were measured by the angles between planes of two aromatic rings.

Table S5 Protein-ligand interactions in M_3R/**3** complex (PDB entry: 4U15) determined by X-ray diffraction (Å, °).

Salt Bridges

Residue	AA	Distance	Protein positive?	Ligand Group[a]
147	ASP	4.49	No	BR

Hydrogen Bonds

Residue	AA	Ligand Group[a]	H–A	D–A	D–H···A
151	SER	BR	1.70	2.49	137.96
507	ASN	Carb	2.15	2.91	129.69

π-cation Interactions

Residue	AA	Ligand Group[a]	Distance[b]
503	TRP	BR	5.57
506	TYR	BR	5.90
529	TYR	BR	4.12

Hydrophobic Interactions

Residue	AA	Ligand Group[a]	Distance
148	TYR	Th	3.76
199	TRP	Th	3.87
225	LEU	Th	3.84
503	TRP	BR	3.76
506	TYR	BR	3.62
529	TYR	BR	3.80

Notes: [a]Ligand group was represented by abbreviation: bicyclic ring (BR), carbonyl group (Carb), thiophenyl group (Th). [b]Distances were measured between centroids determined as the center of aromatic rings.

Table S6 Protein-ligand interactions in M_3R/**4** complex predicted by molecular docking (Å, °).

**Salt Bridges**				
**Residue**	**AA**	**Distance**	**Protein positive?**	**Ligand Group**[a]
147	ASP	4.66	No	BR

**Hydrogen Bonds**					
**Residue**	**AA**	**Ligand Group**[a]	**H–A**	**D–A**	**D–H···A**
507	ASN	Carb	2.67	3.30	119.65

**π-cation Interactions**			
**Residue**	**AA**	**Ligand Group**[a]	**Distance**[b]
148	TYR	BR	5.38
503	TRP	BR	4.91

**Hydrophobic Interactions**			
**Residue**	**AA**	**Ligand Group**[a]	**Distance**
152	ASN	Ph	3.65
155	VAL	Ph	3.98
199	TRP	Ph	3.42
225	LEU	Ph	3.33
231	THR	Ph	3.36
234	THR	Ph	3.30
235	ALA	Ph	3.98
238	ALA	Ph	3.81
503	TRP	BR	3.63
503	TRP	Ph	3.70
529	TYR	BR	3.48
533	TYR	BR	3.46
533	TYR	BR	3.15

Notes: [a]Ligand group was represented by abbreviation: bicyclic ring (BR), carbonyl group (Carb), phenyl group (Ph). [b]Distances were measured between centroids determined as the center of aromatic rings.

Reference

1 Jones, C. G.; Martynowycz, M. W.; Hattne, J.; Fulton, T. J.; Stoltz, B. M.; Rodriguez, J. A.; Nelson, H. M.; Gonen, T. The CryoEM method MicroED as a powerful tool for small molecule structure determination. *ACS Central Science* **2018**, *4* (11), 1587-1592.

2 Hattne, J.; Martynowycz, M. W.; Penczek, P. A.; Gonen, T. MicroED with the Falcon III direct electron detector. *IUCrJ* **2019**, *6* (5), 921-926.

3 Hattne, J.; Reyes, F. E.; Nannenga, B. L.; Shi, D.; De La Cruz, M. J.; Leslie, A. G. W.; Gonen, T. MicroED data collection and processing. *Acta Crystallographica Section A: Foundations and Advances* **2015**, *71* (4), 353-360.

4 Kabsch, W. xds. *Acta Crystallographica Section D: Biological Crystallography* **2010**, *66* (2), 125-132.

5 Kabsch, W. Integration, scaling, space-group assignment and post-refinement. *Acta Crystallographica Section D: Biological Crystallography* **2010**, *66* (2), 133-144.

6 Sheldrick, G. M. SHELXT–Integrated space-group and crystal-structure determination. *Acta Crystallographica Section A: Foundations and Advances* **2015**, *71* (1), 3-8.

7 Sheldrick, G. M. Crystal structure refinement with SHELXL. *Acta Crystallographica Section C: Structural Chemistry* **2015**, *71* (1), 3-8.

8 Hübschle, C. B.; Sheldrick, G. M.; Dittrich, B. ShelXle: a Qt graphical user interface for SHELXL. *Journal of applied crystallography* **2011**, *44* (6), 1281-1284.

9 Datta, N.; Mondal, P.; Pauling, P. The crystal and molecular structure of the acetylcholine antagonist (RS)-4-dimethylamino-2-butynyl 2-cyclohexyl-2-hydroxy-2-phenylacetate hydrochloride. *Acta Crystallographica Section B: Structural Crystallography and Crystal Chemistry* **1979**, *35* (5), 1120-1123.

10 Sládková, V.; Skalická, T.; Skořepová, E.; Čejka, J.; Eigner, V.; Kratochvíl, B. Systematic solvate screening of trospium chloride: discovering hydrates of a long-established pharmaceutical. *CrystEngComm* **2015**, *17* (25), 4712-4721.

11 Pan, X.; Wang, H.; Li, C.; Zhang, J. Z. H.; Ji, C. MolGpka: A web server for small molecule p K a prediction using a graph-convolutional neural network. *Journal of Chemical Information and Modeling* **2021**, *61* (7), 3159-3165.

12 Forli, S.; Huey, R.; Pique, M. E.; Sanner, M. F.; Goodsell, D. S.; Olson, A. J. Computational protein–ligand docking and virtual drug screening with the AutoDock suite. *Nature protocols* **2016**, *11* (5), 905-919.

13 Thorsen, T. S.; Matt, R.; Weis, W. I.; Kobilka, B. K. Modified T4 lysozyme fusion proteins facilitate G protein-coupled receptor crystallogenesis. *Structure* **2014**, *22* (11), 1657-1664.

14 Schrodinger, L. L. C. The PyMOL molecular graphics system. *Version* **2015**, *1*, 8.

15 Liu, Y.; Yang, X.; Gan, J.; Chen, S.; Xiao, Z.-X.; Cao, Y. CB-Dock2: Improved protein–ligand blind docking by integrating cavity detection, docking and homologous template fitting. *Nucleic acids research* **2022**, *50* (W1), W159-W164.

16 Eberhardt, J.; Santos-Martins, D.; Tillack, A. F.; Forli, S. AutoDock Vina 1.2. 0: New docking methods, expanded force field, and python bindings. *Journal of chemical information and modeling* **2021**, *61* (8), 3891-3898.
17 Trott, O.; Olson, A. J. AutoDock Vina: improving the speed and accuracy of docking with a new scoring function, efficient optimization, and multithreading. *Journal of computational chemistry* **2010**, *31* (2), 455-461.
18 Adasme, M. F.; Linnemann, K. L.; Bolz, S. N.; Kaiser, F.; Salentin, S.; Haupt, V. J.; Schroeder, M. PLIP 2021: Expanding the scope of the protein–ligand interaction profiler to DNA and RNA. *Nucleic acids research* **2021**, *49* (W1), W530-W534.
19 Kaduk, J. A.; Boaz, N. C.; Gindhart, A. M.; Blanton, T. N. Crystal structure of oxybutynin hydrochloride hemihydrate, $C_{22}H_{32}NO_3Cl\ (H_2O)_{0.5}$. *Powder Diffraction* **2019**, *34* (1), 50-58.
20 Macrae, C. F.; Sovago, I.; Cottrell, S. J.; Galek, P. T. A.; McCabe, P.; Pidcock, E.; Platings, M.; Shields, G. P.; Stevens, J. S.; Towler, M. Mercury 4.0: From visualization to analysis, design and prediction. *Journal of applied crystallography* **2020**, *53* (1), 226-235.
